# Cutaneous zosteriform endometrial cancer metastasis

**DOI:** 10.1111/1346-8138.17536

**Published:** 2024-11-27

**Authors:** Wangxiang Luo, Xianhong Yang, Yunlin Ren, Xiaohua Tao, Wei Lu

**Affiliations:** ^1^ Department of Dermatology, Center for Plastic & Reconstructive Surgery Zhejiang Provincial People's Hospital (Affiliated People's Hospital, Hangzhou Medical College) Hangzhou China; ^2^ Department of Dermatology Yiwu Skin Disease Hospital Jinhua China

**Keywords:** cutaneous metastases, endometrial cancer, zosteriform

A 58‐year‐old woman sought care for painful skin papules and blisters on her right lower extremity, ongoing for a month. Initially diagnosed with herpes zoster, she received no relief from famciclovir or acyclovir treatments. The lesions spread to her thighs, manifesting as itchy, tingling spots. With a history of poorly differentiated endometrial cancer for 11 years, she had undergone radical surgery and multiple chemotherapy regimens. Systemic examination revealed left cervical lymph node enlargement. Skin inspection showed swelling and redness with bumps on her right lower leg and left thigh base (referenced in Figure 1a,b). A thigh biopsy showed tumor cells arrayed among dermal collagen in various sizes with intense chromatin and clear nuclear division (referenced in Figure 1c,d). The dermal collagen was markedly sclerotic with few inflammatory cells. Immunohistochemistry was positive for P53, EMA, and PAX8, and D2‐40 staining indicated lymphatic infiltration by tumor cells. She was diagnosed with cutaneous metastasis from poorly differentiated endometrial adenocarcinoma.

**Figure 1:**
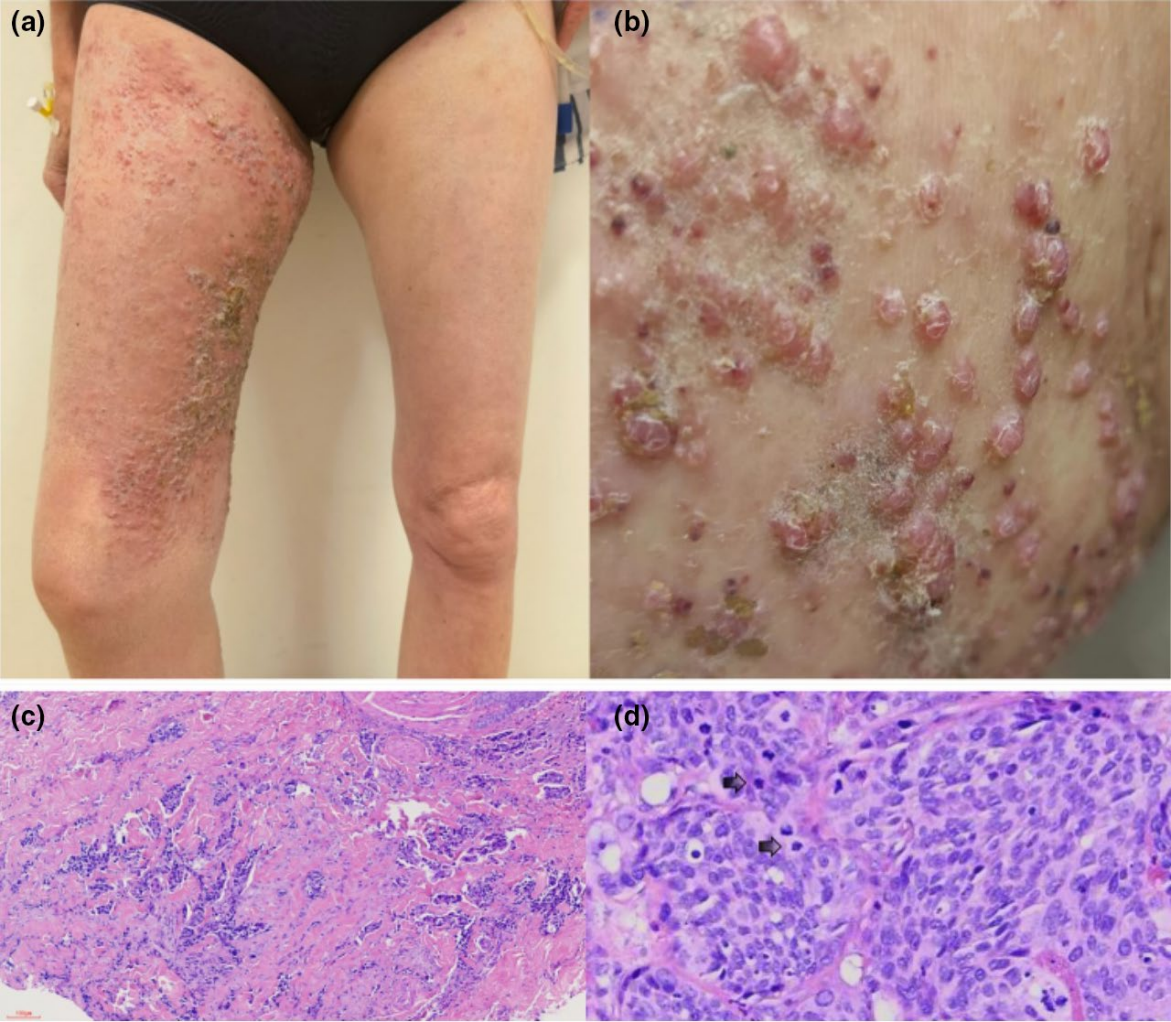
(a, b) Metastatic endometrial carcinoma presented with erythema, papules, and blisters across the right thigh and base of the left thigh, blending into the surrounding skin without clear demarcation. (c) Sclerotic dermal collagen fibers with a reddish hue and rigidity, sparsely populated by inflammatory cells; original magnification, ×100. (d) At 400× magnification, tumor cells displayed varied sizes, pronounced chromatin, intense staining, and noticeable nuclear pleomorphism.

Cutaneous metastasis from carcinoma is a rare event in the spread of cancer, seen in 0.7%–9% of metastatic cases. It is even rarer in endometrial cancer, with an incidence of less than 0.8%.^1^ Zosteriform metastasis in skin cancer is a rare phenomenon linked to malignancies such as lung, breast, and gastric cancers, and melanoma. It typically manifests in a band‐like pattern, often associated with pain, indicating disease progression and correlating with the primary tumor's location.^2^

In this case, the patient was diagnosed with endometrial cancer. When skin metastasis was subsequently discovered, the patient had already entered the late stage of malignant tumor treatment, with a poor prognosis. Therefore, when patients with pre‐existing endometrial cancer suddenly present with new skin lesions, such as shingles, they need to be alert to the possibility of skin metastasis.

## CONFLICT OF INTEREST STATEMENT

None declared.
